# Unveiling the role of Pleckstrin-2 in tumor progression and immune modulation: insights from a comprehensive pan-cancer analysis with focus on lung cancer

**DOI:** 10.1186/s43556-024-00225-8

**Published:** 2024-11-15

**Authors:** Enzhi Yin, Chengming Liu, Yuxin Yao, Yuejun Luo, Yaning Yang, Xiaoya Tang, Sufei Zheng, Linyan Tian, Jie He

**Affiliations:** 1 Department of Thoracic Surgery, National Cancer Center/National Clinical Research Center for Cancer/Cancer Hospital, Chinese Academy of Medical Sciences and Peking Union Medical College, Beijing, 100021 China; 2 State Key Laboratory of Molecular Oncology, National Cancer Center/National Clinical Research Center for Cancer/Cancer Hospital, Chinese Academy of Medical Sciences and Peking Union Medical College, Beijing, 100021 China; 3 Department of Medical Oncology, National Cancer Center/National Clinical Research Center for Cancer/Cancer Hospital, Chinese Academy of Medical Sciences and Peking Union Medical College, Beijing, 100021 China

**Keywords:** PLEK2, Pan-cancer, Biomarker, Prognosis, Immunotherapy

## Abstract

Cancer remains a leading cause of mortality globally, highlighting the need for novel biomarkers to enhance prognosis and therapeutic strategies. Pleckstrin-2 (PLEK2), a member of the pleckstrin family, has been implicated in processes critical to tumor progression, but its role across cancers remains underexplored. This study systematically examined the expression patterns, prognostic relevance, and functional impact of PLEK2 across multiple cancer types. Using data from The Cancer Genome Atlas (TCGA), Genotype Tissue Expression Project (GTEx), and the Human Protein Atlas, we analyzed PLEK2 expression in both cancerous and normal tissues, revealing significant overexpression of PLEK2 in various cancers at the mRNA and protein levels. Single-cell RNA sequencing further indicated predominant expression of PLEK2 in tumor cells and macrophages within the tumor microenvironment. Survival analysis demonstrated that elevated PLEK2 expression correlated with poor prognosis in specific cancers, though its impact varied across cancer types. Functional assays showed that PLEK2 knockdown inhibited proliferation and migration in human cancer cell lines. In vivo studies using a Lewis lung carcinoma (LLC) model confirmed that PLEK2 knockdown suppressed tumor growth and enhanced the efficacy of PD-1 immunotherapy. Mechanistically, PLEK2 knockdown was associated with reduced AKT pathway activation, diminished tumor-associated macrophage infiltration, and increased CD8 T cell presence. Compounds like Navitoclax were also identified as potential PLEK2 inhibitors. In conclusion, PLEK2 played a multifaceted role in cancer progression and immune response modulation. Targeting PLEK2 might suppress tumor growth and overcome immunotherapy resistance, offering a promising biomarker and therapeutic target to improve cancer treatment outcomes.

**Supplementary Information:**

The online version contains supplementary material available at 10.1186/s43556-024-00225-8.

## Introduction

Cancer remains a significant global health challenge, causing approximately 9.96 million deaths in 2020. It stands as a leading cause of mortality among individuals under 70 years of age in 112 out of 183 countries [1, 2]. The diverse nature of cancer biology and its complex interplay with host immunity have led to continuous efforts to develop effective therapies. Immunotherapy, especially approaches like Chimeric Antigen Receptor T cell (CAR-T) therapy and immune checkpoint blockade (ICB), has brought about a paradigm shift in cancer treatment [3, 4]. These innovations have shown remarkable success in some cancers, significantly improving patient survival rates. Despite these advances, many challenges remain, including limited response rates, varying efficacy across different tumor types, the emergence of resistance, and significant adverse effects such as autoimmune complications [5]. This underscores the need for further research to identify new biomarkers that can optimize immunotherapy by predicting which patients will benefit from specific treatments [6], thereby reducing unnecessary toxicity and healthcare costs [7].

Pleckstrin-2 (PLEK2), a member of the Pleckstrin family, is known to regulate actin cytoskeleton dynamics by promoting the formation of lamellipodia and peripheral ruffles, which are structures critical for cell motility [8]. Increasing evidence suggests that PLEK2 plays a role in the progression of various cancers. Our previous studies highlighted that PLEK2 contributes to metastasis and chemoresistance in esophageal squamous cell carcinoma (ESCC) [9]. Similarly, elevated PLEK2 expression has been associated with enhanced invasion and metastasis in multiple cancers, including gallbladder cancer [10], non-small cell lung cancer [11], breast cancer [12], and head and neck squamous cell carcinoma (HNSCC) [13]. In addition, recent findings suggest a potential relationship between PLEK2 expression and immune cell infiltration in the tumor microenvironment, particularly in gastric cancer (GC) and ESCC [14, 15]. These observations suggest that PLEK2 might play a multifaceted role in shaping both tumor behavior and immune interactions.

However, despite these insights, there remains a notable gap in the systematic exploration of PLEK2’s role across different cancer types. A comprehensive pan-cancer analysis is lacking, particularly one that investigates the prognostic significance of PLEK2 and its potential to predict responses to immunotherapy. Understanding how PLEK2 influences tumor progression and immune responses could provide valuable insights for enhancing therapeutic strategies. Given the heterogeneous nature of cancer and the variability in responses to immunotherapy, elucidating the role of PLEK2 across multiple tumor types could aid in the identification of novel therapeutic targets and improve the precision of immunotherapy.

In this study, we performed an extensive analysis of PLEK2 across various cancers using publicly available datasets, including The Cancer Genome Atlas (TCGA) and Gene Expression Omnibus (GEO). We evaluated the differential expression of PLEK2 in cancerous and normal tissues, explored its impact on immune cell infiltration, and assessed its potential as a predictive biomarker for patient prognosis and response to immune checkpoint blockade therapy. We also undertook an analysis to identify candidate compounds that may target PLEK2. To validate these findings, in vitro experiments were conducted across nine cancer cell lines, and in vivo studies were performed using the Lewis lung carcinoma (LLC) bearing mouse model. The choice of LLC as a model allowed us to study the role of PLEK2 in an immunocompetent environment, particularly its influence on enhancing immunotherapy efficacy. The outcomes of our study are expected to elucidate the potential role of PLEK2 in tumor immunology and offer fresh insights for the advancement of immunotherapy research.

## Results

### Expression of *PLEK2* in multiple cancers

**Fig. 1:**
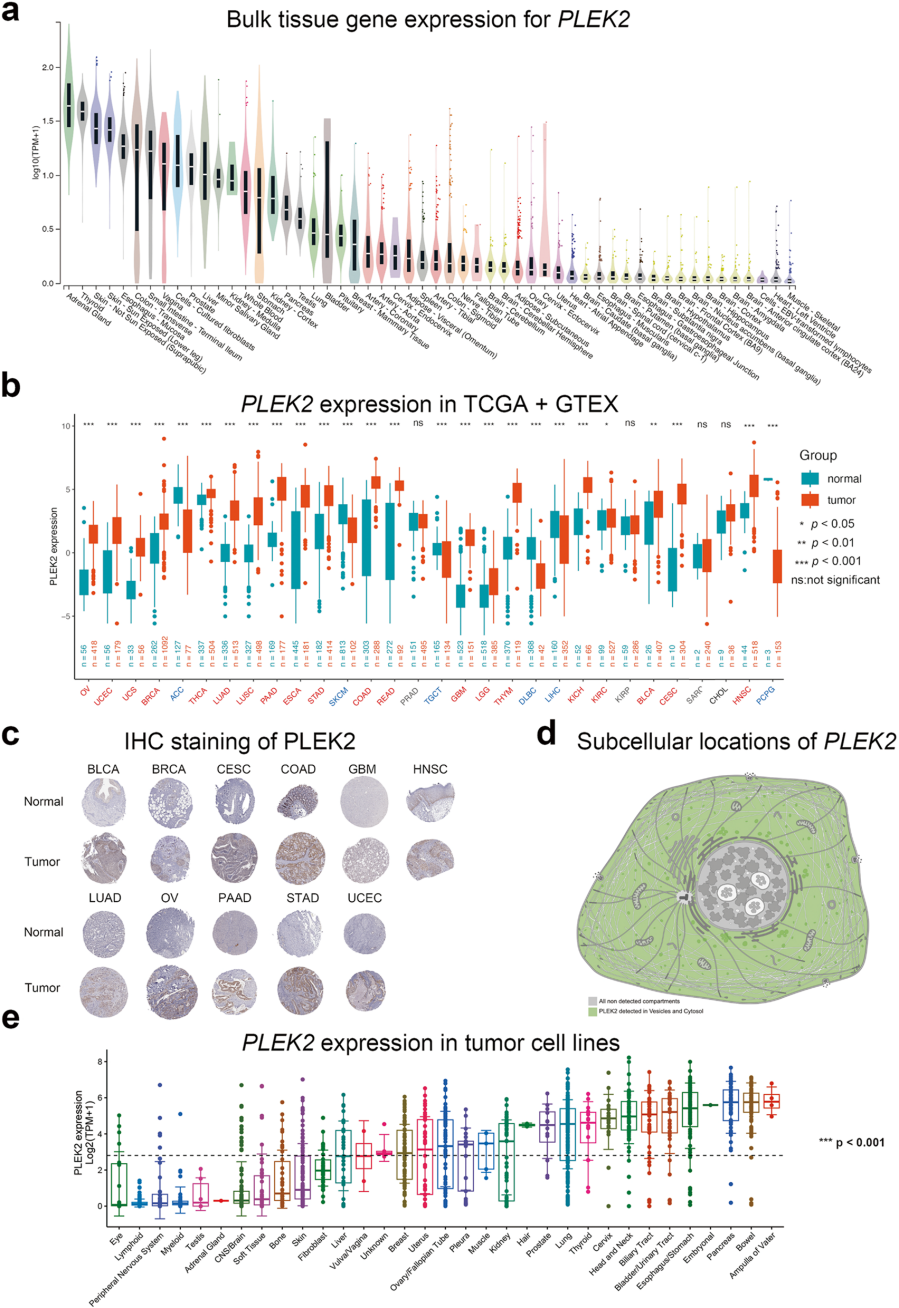
mRNA expression levels of PLEK2 in pan-cancer analysis. **a** Violin plots showing *PLEK2* expression levels in various human normal tissues. **b** Boxplots illustrating the mRNA expression levels of *PLEK2* in normal and cancer tissues using data from the TCGA and GTEx databases. Tumors that were not statistically significant were labeled in black, those with significantly elevated *PLEK2* expression compared to normal tissue were labeled in red, and those with reduced considerably were labeled in blue. **c** Representative images of immunohistochemical (IHC) staining of PLEK2 in 11 types of normal and tumor tissues. **d** Subcellular locations of PLEK2 from the HPA database. The confidence scale is color-coded, ranging from light green (1) for low confidence to dark green (5) for high confidence. Gray (0) indicates an absence of localization evidence. **e** The expression level of the *PLEK2* in tumor cell lines

The study design flowchart is depicted in Figure S1. To investigate *PLEK2* expression patterns across various cancers, we first examined its basal expression in normal human tissues using the GTEx database [16]. As shown in Fig. 1a, *PLEK2* mRNA is notably elevated in multiple organs prone to cancer development, while lower expression levels are observed in non-proliferative tissues like the heart and brain. We then compared *PLEK2* mRNA levels between cancerous and normal tissues, compiling data from 30 cancer types in the TCGA and GTEx databases. This analysis revealed significantly elevated *PLEK2* expression in most cancers, except for ACC, SKCM, TGCT, DLBC, LIHC, and PCPG (Fig. 1b, normal vs. tumor, *P* < 0.05, Student’s t-test). To validate these findings at the protein level, we utilized immunohistochemistry (IHC) data from the Human Protein Atlas (HPA) [17]. As illustrated in Fig. 1c, *PLEK2* protein is significantly upregulated in several cancers compared to normal tissues, including BLCA, BRCA, CESC, COAD, GBM, HNSC, LUAD, OV, PAAD, STAD, and UCEC. Corresponding clinical information for these samples is provided in Figure S2. We also examined the subcellular localization of *PLEK2* using HPA data, finding it predominantly localized in vesicles and the cytosol (Fig. 1d). Additionally, analysis of cancer cell lines indicated elevated *PLEK2* expression in specific cancer types, such as malignant neoplasms of the digestive system, bladder, head and neck, cervix, thyroid, lung, pleural mesothelioma, and breast cancer (Fig. 1e). Overall, these findings suggested that *PLEK2* was overexpressed in various cancers and might play a significant role in tumorigenesis, highlighting its potential as a clinical diagnostic marker.

### Single-cell expression levels of *PLEK2* in multiple tumor tissues

**Fig. 2:**
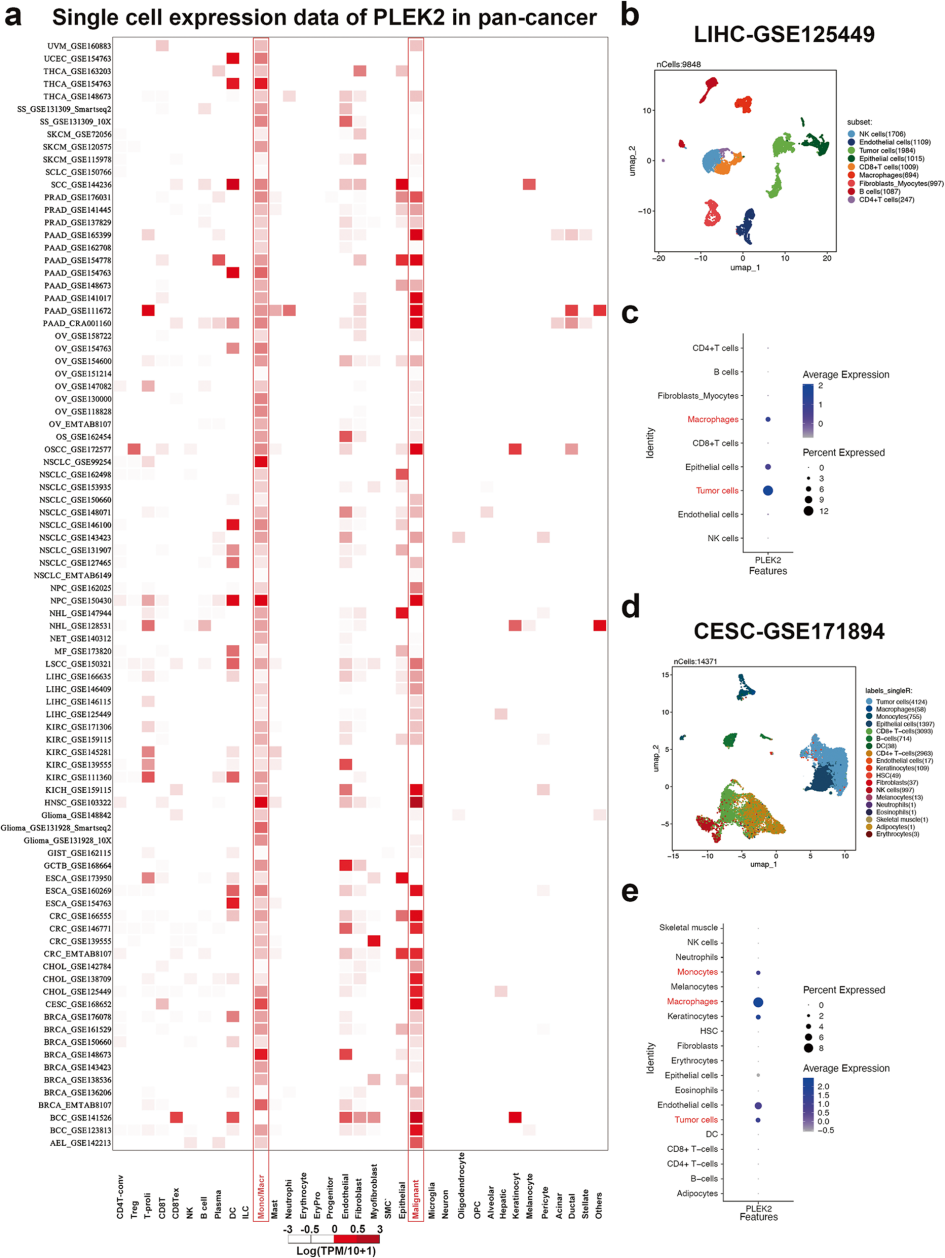
Single-cell expression analysis of *PLEK2* across cancers. **a** Cluster heatmaps showing the mRNA levels of *PLEK2* in various tumors. **b, d** Umap plots displaying the clustering of different cell types in LIHC (**b**) and CESC (**d**). **c, e** Bubble diagram showing *PLEK2* expression level in LIHC (**c**) and CESC (**e**)

To determine the specific cell types expressing *PLEK2* in tumor tissues, we analyzed its single-cell expression across 88 datasets using the Tumor Immune Single-cell Hub (TISCH) online tool [18]. As shown in Fig. 2a, the heatmap illustrates the relative expression levels of *PLEK2* across 35 cell types, indicating its widespread presence in various immune and malignant cells. Notably, *PLEK2* exhibited higher expression levels in tumor cells and immune cells such as macrophages and monocytes. To validate these findings, we downloaded the LIHC dataset (LIHC_GSE125449) from TISCH and three additional datasets not included in TISCH—CESC_GSE171894, STAD_GSE183904, and ESCA_GSE188900—for manual single-cell analyses. In the LIHC_GSE125449 dataset, Figs. 2b and c display *PLEK2* expression in CD4 T cells, CD8 T cells, NK cells, and B cells, with particularly high expression observed in macrophages. Similarly, in the other three datasets, *PLEK2* was predominantly expressed in tumor cells and monocytes/macrophages, as shown in Figs. 2d, e, and Figure S3. Consistently across all datasets, the elevated expression of *PLEK2* in monocytes/macrophages and tumor cells suggested that *PLEK2* might play a significant role in tumor immunity.

### Prognostic significance of *PLEK2* in pan-cancer

**Fig. 3:**
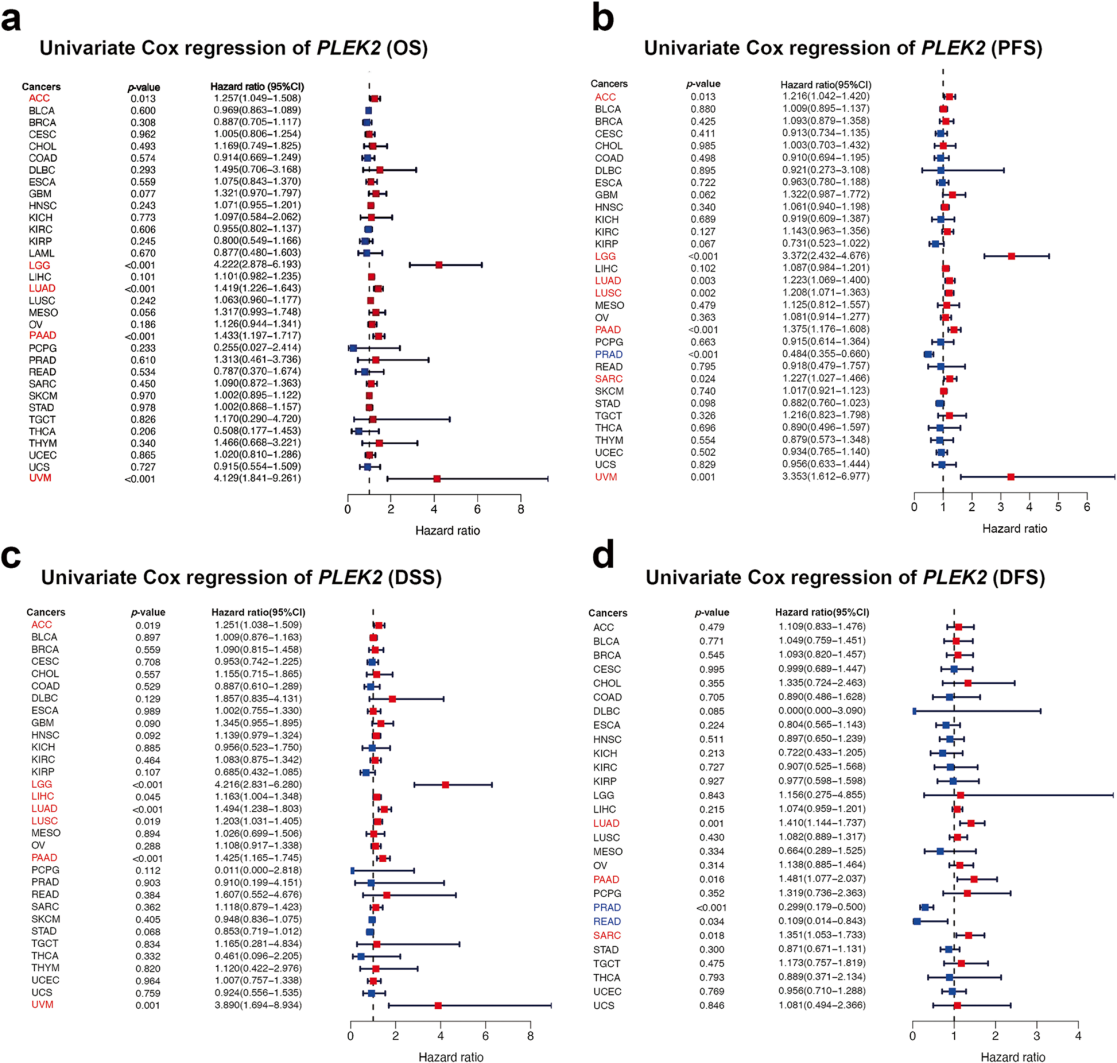
Association between *PLEK2* expression levels and prognosis from the TCGA database. **a** A forest plot depicting the correlation between *PLEK2* expression and OS across 33 different tumor categories. **b** A forest plot illustrating the relationship between the expression of *PLEK2* and PFS in 33 tumor types. **c** A forest plot showing the connection between *PLEK2* expression and DSS in 33 varieties of tumors. **d** A forest plot detailing the association between *PLEK2* expression and DFS across 33 tumor categories. Red indicated that *PLEK2* was a risk factor affecting the prognosis of cancer patients, and blue represents a protective factor

To assess the prognostic value of *PLEK2* across various cancers, we performed survival association analyses including overall survival (OS), progression-free survival (PFS), disease-specific survival (DSS), and disease-free survival (DFS). Elevated expression of *PLEK2* was significantly correlated with reduced OS, PFS, DSS, and DFS in LUAD and PAAD (Fig. 3a–d). Specifically, higher *PLEK2* expression was associated with unfavorable OS in ACC, LGG, and UVM (Fig. 3a). The forest plot analyses indicated that elevated *PLEK2* expression corresponded to shortened PFS in ACC, LGG, LUSC, SARC, and UVM (Fig. 3b). Additionally, increased *PLEK2* expression was linked to poorer DSS in ACC, LGG, LIHC, LUSC, and UVM (Fig. 3c). Cox proportional hazards model analysis revealed that *PLEK2* expression levels were significantly associated with DFS in PRAD, READ, and SARC (Fig. 3d). Notably, this analysis suggested that *PLEK2* might serve as a protective factor for PRAD and READ in PFS and DFS outcomes (Fig. 3b). The association between *PLEK2* expression and clinical features in these cancers was also resolved and displayed in Figure S4. Further analysis using Kaplan–Meier survival curves demonstrated that lower expression of *PLEK2* was related to better OS in ACC, GBM, LGG, LIHC, LUAD, MESO, OV, PAAD, SARC, SKCM, and UVM (Figures S5a, i, o and S6a, b, d, e, f, j, k, and r, respectively). Conversely, high *PLEK2* expression was associated with favorable prognosis in BLCA, BRCA, COAD, DLBC, HNSC, KIRP, PCPG, PRAD, READ, and THCA (Figures S5b, c, f, g, j, m and S6g, h, i, n, respectively). These findings indicated that *PLEK2* played a prognostic role in predicting cancer outcomes; however, its impact varied across different cancer types, suggesting a complex and multifaceted role in cancer progression.

### Predictive potential of *PLEK2* in cancer immunotherapy response

**Fig. 4:**
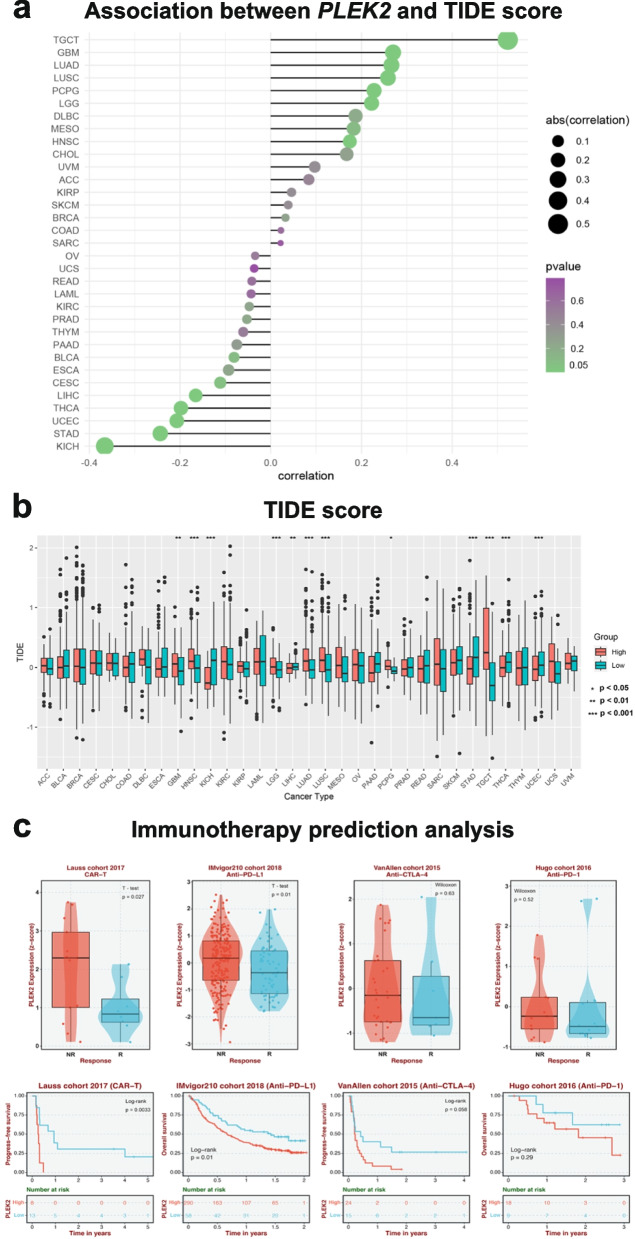
The association of *PLEK2* expression with immunotherapy response and Tumor Immune Dysfunction and Exclusion (TIDE) scores. **a** The association between *PLEK2* expression and TIDE score. **b** The distribution of TIDE scores across *PLEK2* high and low expression groups in pan-tumors. **c** The expression of *PLEK2* in response and non-response groups of various immunotherapeutic cohorts

Building on our single-cell analysis indicating that *PLEK2* is expressed in various immune cells, particularly macrophages (Fig. 2a), we aimed to investigate the predictive role of *PLEK2* expression in cancer immunotherapy responses across multiple cancer types. To assess this potential, we calculated Tumor Immune Dysfunction and Exclusion (TIDE) scores—a well-established biomarker for predicting immunotherapy outcomes—for patients stratified by *PLEK2* expression levels [19–22]. Our analysis revealed that TIDE scores positively correlated with *PLEK2* expression across several malignancies, notably in TGCT, GBM, LUAD, LUSC, PCPG, LGG, MESO, and HNSC (Fig. 4a). Specifically, In GBM, HNSC, LGG, LUAD, LUSC, PCPG, and TGCT, high *PLEK2* expression correlated with higher TIDE scores, suggesting these patients may derive less benefit from immunotherapy. Conversely, in KICH, LIHC, STAD, THCA, and UCEC, patients with low *PLEK2* expression exhibited higher TIDE scores, also indicating a reduced benefit from immunotherapy (Fig. 4b). In addition, we examined *PLEK2* expression levels across different cohorts undergoing immunotherapy, including those treated with anti-PD-1/PD-L1, anti-CTLA-4, and CAR-T cell therapies. Our findings indicated that elevated *PLEK2* expression correlated with a lack of response to immunotherapy, as patients in the non-responder groups showed higher *PLEK2* levels than those who responded (Fig. 4c). These findings collectively implied that elevated *PLEK2* expression could be strongly linked to resistance against immunotherapy in certain cancers.

### Association between *PLEK2* and immunological characteristics across cancers

**Fig. 5:**
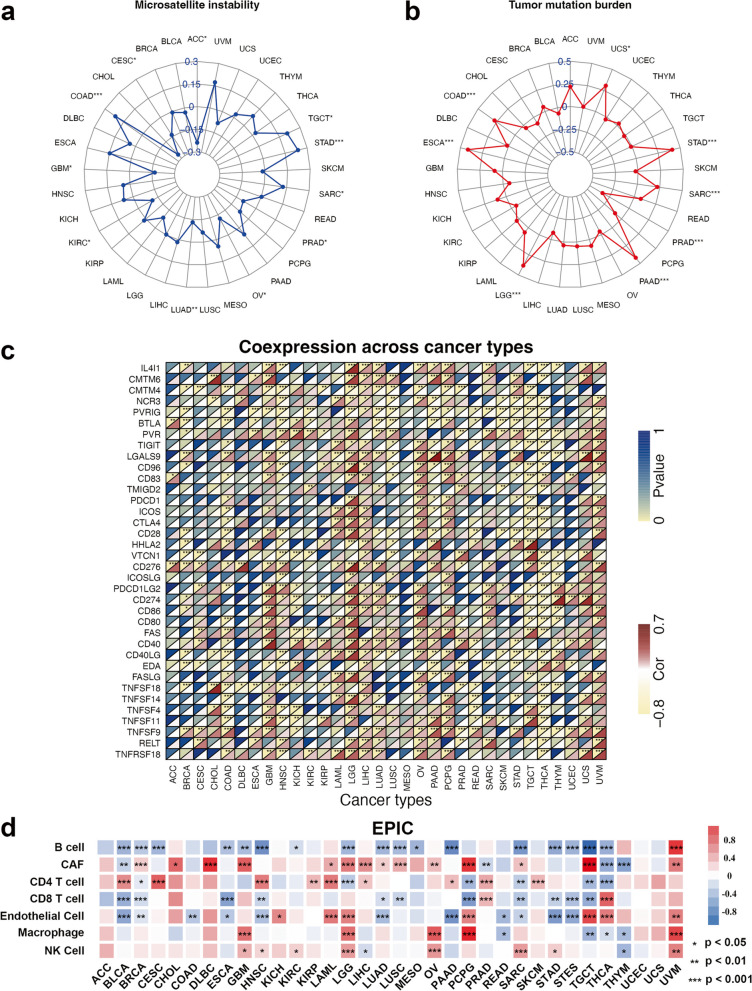
Relationship between *PLEK2* expression and various immune characteristics. **a** Correlation between *PLEK2* expression and MSI displayed by the radar chart. **b** Correlation between *PLEK2* expression and TMB displayed by the radar chart. **c** Relationship between *PLEK2* expression and various immune checkpoints. **d** *PLEK2* was closely related to the immune infiltration level in multiple tumor tissues analyzed via EPIC algorithms

Considering the significance of programmed death-ligand 1 (PD-L1) [23], tumor mutation burden (TMB) [24], and microsatellite instability (MSI) as important biomarkers for immunotherapy response [25], we assessed the correlation between *PLEK2* expression and TMB/MSI across multiple cancer types. *PLEK2* expression was negatively associated with high MSI scores in ACC, CESC, GBM, KIRC, LUAD, OV, and PRAD. Conversely, it was positively associated with high MSI scores in COAD, SARC, STAD, and TGCT (Fig. 5a). Moreover, negative correlations between *PLEK2* expression and TMB were identified in PRAD, while positive correlations were observed in COAD, ESCA, LGG, PAAD, SARC, STAD, and UCS (Fig. 5b). These results suggested that *PLEK2* might serve as a predictive marker for the efficacy of cancer immunotherapy in these cancers.

Immune-related regulators play a vital role in shaping the tumor microenvironment and determining the success of cancer immunotherapy [26]. We explored the relationship between *PLEK2* expression and various immunomodulatory molecules, focusing on members of the B7-CD28 family, tumor necrosis factor (TNF) family, and both established and emerging immune checkpoints. Our analysis revealed that elevated *PLEK2* expression was significantly associated with increased expression of several key immune checkpoint molecules, such as CD276 (B7-H3), CD274 (PD-L1), LGALS9, PVR (CD155), and FAS across a wide spectrum of cancers (Fig. 5c).

To investigate whether *PLEK2* expression influences immune cell infiltration in human cancers, we performed Estimating the Proportions of Immune and Cancer cells (EPIC) analysis to compare immune scores between patients with high and low *PLEK2* expression across 34 cancer types. As shown in Fig. 5d, *PLEK2* expression was positively associated with the infiltration of cancer-associated fibroblasts (CAFs), CD4 T cells, macrophages, and natural killer (NK) cells, but negatively associated with CD8 T cells and B cells. These findings indicated that *PLEK2* expression in tumor cells might regulate the infiltration and exhaustion of immune cells, thereby impacting the prognosis and response to immunotherapy in human cancers.

### GSEA analysis of *PLEK2* and its implications for immunotherapy and drug sensitivity prediction

**Fig. 6:**
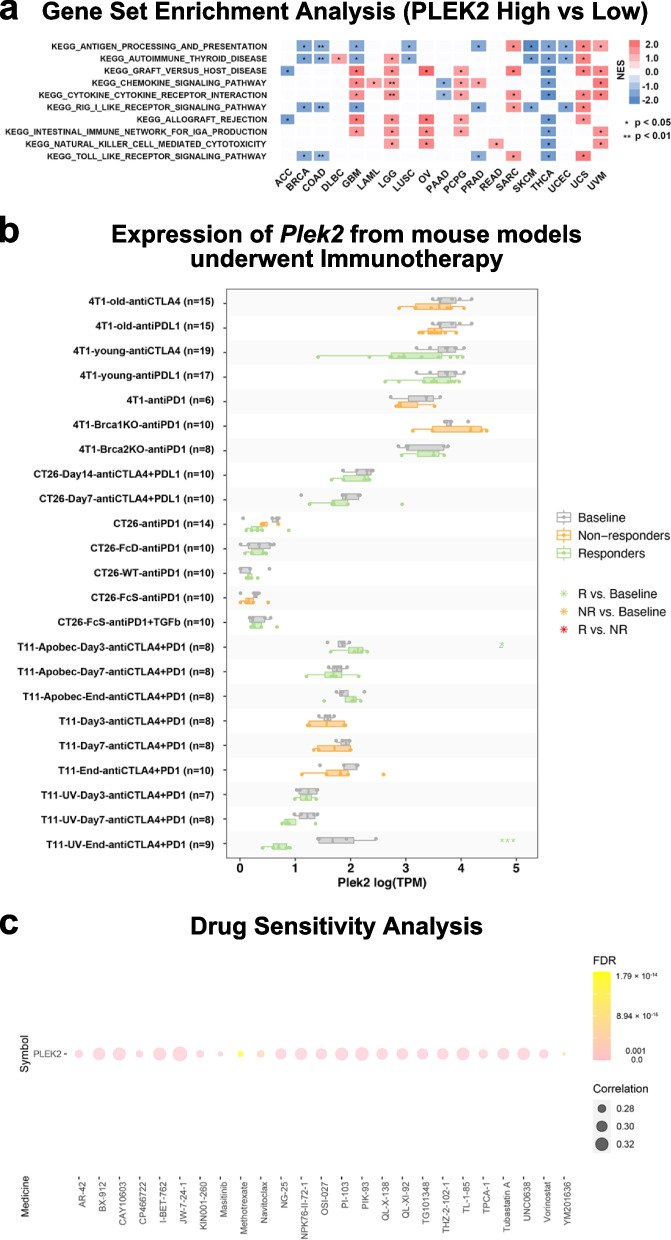
Relationship between *PLEK2* expression and immune-related pathways and drug sensitivity analysis. **a** Top 10 enriched immune-related pathways based on the KEGG terms. **b** Multiple boxplots depict the expression of *Plek2* in tumors from mouse models before and after PDL1 or CTLA4 treatment, as obtained from the TISMO web tool. Tumor models included: Mammary cancer: 4T1 and T11; Colorectal carcinoma: CT26. **c** Based on drug sensitivity analysis in GDSC, the top 25 drugs that are positively correlated with *PLEK2* expression are shown in the figure

To further elucidate the influence of *PLEK2* on tumor immunity, we conducted a pan-cancer Gene Set Enrichment Analysis (GSEA). Our results revealed distinct expression patterns in immune-related pathways, particularly those involved in antigen processing and presentation, autoimmune thyroid disease, RIG-I-like receptor signaling, and Toll-like receptor signaling pathways (Fig. 6a). These pathways were negatively enriched in patients with high *PLEK2* expression in several cancers, including BRCA, COAD, LUSC, PRAD, SKCM, THCA, and UCEC. These findings suggested that elevated *PLEK2* expression might contribute to suppressing antitumor immune responses within these malignancies, implicating *PLEK2* in the modulation of tumor-immune interactions and potentially impacting the efficacy of immune-based therapies.

Recognizing the pivotal role of animal studies in understanding the mechanisms behind tumor immunotherapy [27, 28], we explored the prognostic capabilities of *Plek2* in mouse models using the TISMO database [29]. Our analysis revealed that *Plek2* expression levels were indicative of how mouse models of 4T1, T22 (mammary cancer), and CT26 (colorectal carcinoma) responded to immunotherapy treatments targeting PD-L1 and CTLA-4 (Fig. 6b). Specifically, higher *Plek2* expression correlated with reduced efficacy of these immunotherapies, suggesting a potential role for *Plek2* in mediating immunotherapy resistance. To identify potential strategies to mitigate the tumor-enhancing effects of *Plek2*, we assessed drug sensitivity associated with its expression. Analysis of the Genomics of Drug Sensitivity in Cancer (GDSC) dataset identified Navitoclax, Methotrexate, and YM201636 as the top compounds showing a positive correlation with *PLEK2* expression (Fig. 6c) [30]. These drugs might be more effective in tumors with high *PLEK2* expression, offering potential therapeutic avenues. These findings underscored the significant impact of *Plek2* on the effectiveness of immunotherapeutic interventions in mouse models.

### *PLEK2* knockdown suppressed cell proliferation and migration in multiple cancer cells

**Fig. 7:**
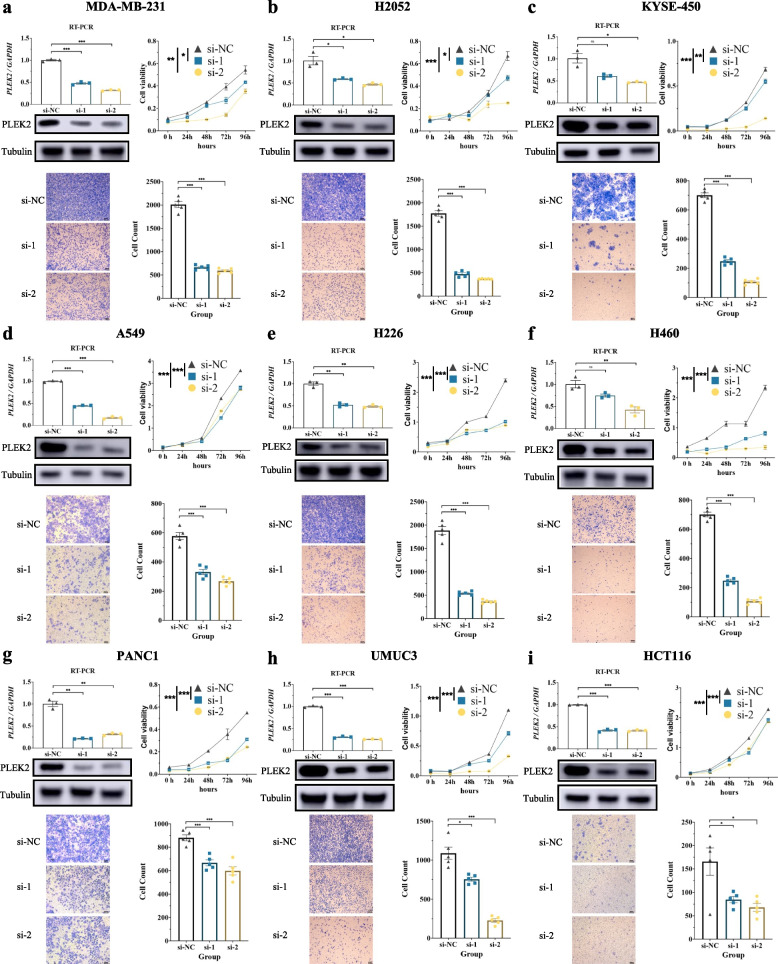
Silence of *PLEK2* inhibits cell proliferation and cell migration on 9 cancer cell lines. **a-i** Top left panels show RT-PCR and WB verification of the silent efficiency of *PLEK2* in MDA-MB-231 (**a**), H2052 (**b**), KYSE-450 (**c**), A549 (**d**), H226 (**e**), H460 (**f**), PANC-1 (**g**), UM-UC-3 (**h**) and HCT116 cells (**i**); *n* = 3/group. CCK-8 assays evaluated cellular growth curves in top right; *n* = 6/group. Representative images (bottom left) and quantification of colony formation assays (bottom right) of indicated cells transfected with si*PLEK2*; *n* = 5/group. All experiments were repeated three times. Data are presented as mean ± SEM. Data were analyzed by one-way ANOVA

To further elucidate the functional role of *PLEK2* in tumor cells, we performed transient siRNA-mediated knockdown of *PLEK2* in various human cancer cell lines. Specifically, we targeted *PLEK2* in MDA-MB-231 (BRCA), H2052 (MESO), KYSE-450 (ESCA), A549 (LUAD), H226 (LUSC), H460 (large cell lung carcinoma), PANC-1 (PAAD), UM-UC-3 (BLCA), and HCT-116 (COAD) cell lines. The efficiency of *PLEK2* knockdown was verified by quantitative real-time PCR (qRT-PCR) and Western blot analysis (Fig. 7a-i top left). Among the two siRNA constructs tested, Si2 demonstrated superior knockdown efficiency compared to Si1, as evidenced by qRT-PCR results. We assessed the impact of *PLEK2* knockdown on cell proliferation using appropriate assays and observed a significant inhibition of proliferation in seven of the nine cell lines tested (Fig. 7a-c and e–h). Notably, in the A549 and HCT-116 cell lines (Fig. 7d and i), *PLEK2* knockdown resulted in only a slight reduction in cell proliferation. Furthermore, cell migration assays revealed that *PLEK2* knockdown markedly inhibited the migration of all nine cancer cell lines (Fig. 7a-i bottom). These findings underscored that downregulation of *PLEK2* hampered cellular proliferation and migration across multiple cancer types.

### *Plek2* promoted tumor growth and influenced the efficacy of PD-1 immunotherapy in LLC model

**Fig. 8:**
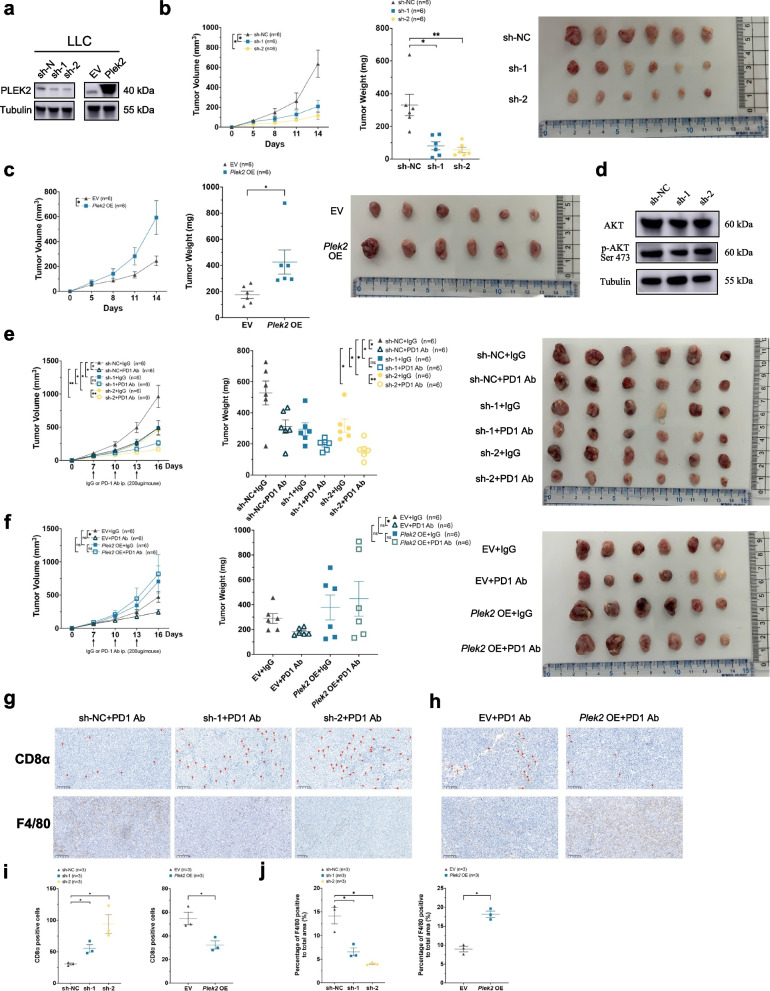
*Plek2* promoted tumor growth and influenced the efficacy of PD-1 immunotherapy in Lewis Lung Carcinoma (LLC) model. **a** Western blot confirming *Plek2* knockdown (sh-1, sh-2) and overexpression (*Plek2* OE) in LLC cells. **b** Plek2 knockdown LLC cells (sh-1, sh-2) or control (sh-NC) were subcutaneously injected into nude mice. Tumor size was measured every 3 days (**b** left panel), and after 14 days, tumors were dissected and analyzed for tumor weight (**b** middle panel) and tumor grafts (**b** right panel). *n* = 6/group. **c** *Plek2* overexpression (*Plek*2 OE) or empty vector (EV) LLC cells were injected into nude mice. Tumor size (**c** left panel), tumor weight (**c** middle panel), and tumor grafts (**c** right panel) were evaluated 14 days post-transplantation. *n* = 6/group. **d** Western blot of AKT and phospho-AKT (Ser 473) levels in *Plek2* knockdown LLC cells (dsh-1, sh-2) or control (sh-NC), with tubulin as a loading control. **e** C57BL/6 mice were injected with *Plek2* knockdown or control LLC cells. Tumor size (**e** left panel), tumor weight (**e** middle panel), and tumor grafts (**e** right panel) were measured 16 days post-transplantation. PD-1 antibody or isotype control IgG2a was administered (indicated by arrows). *n* = 6/group. f *Plek2* overexpression or control cells were injected into C57BL/6 mice, and tumor size (**f** left panel), tumor weight (**f** middle panel), and tumor grafts (**f** right panel) were assessed. PD-1 antibody or IgG2a control was administered (arrows). *n* = 6/group. **g, h** Representative immunohistochemistry of CD8α (indicated by red arrows) and F4/80 in LLC tumor tissues after PD-1 antibody treatment, following *Plek2* knockdown (**g**) or overexpression (**h**). Magnification 400 × , scale bar = 100 μm. **i, j** Quantification of CD8α cells (**i**) and F4/80 area (**j**). *n* = 3/group. Data are presented as mean ± SEM

To further validate the role of PLEK2 in in vivo experiments, we chose to construct an animal model using a lung cancer cell line for the following reasons: 1. Lung cancer remains one of the leading causes of cancer-related mortality worldwide, highlighting the urgent need for improved biomarkers and therapeutic targets [31]. 2. Our previous bioinformatics analysis showed that high *PLEK2* expression was closely associated with poor prognosis in LUAD patients (Fig. 3), and it correlated with lower TIDE scores (Fig. 4a), indicating a potential role for *PLEK2* in immune evasion. 3. In our in vitro experiments, we used three non-small cell lung cancer (NSCLC) cell lines to confirm that *PLEK2* knockdown significantly inhibited tumor cell growth and migration (Fig. 7d-f). We generated stable *Plek2* knockdown and overexpression LLC cell lines (Fig. 8a) and conducted in vitro assays to assess the impact of *Plek2* modulation on proliferation and migration. Consistent with our findings in nine human cancer cell lines, *Plek2* knockdown significantly inhibited both proliferation and migration of LLC cells (Figures S7a and S7c), while *Plek2* overexpression enhanced these abilities (Figures S7b and S7d). These results confirmed that *Plek2* promoted cellular proliferation and migration in LLC cells. To further validate the role of *Plek2* in tumor growth, we conducted in vivo experiments by implanting LLC cells into BALB/c nude mice. As shown in Fig. 8b, *Plek2* knockdown significantly reduced tumor growth rates and tumor weights by day 14 after transplantation, while *Plek2* overexpression markedly increased both (Fig. 8c). These findings underscored *Plek2*’s crucial role in tumor development and progression.

In previous studies, we found that *PLEK2* knockdown inhibited proliferation and migration in esophageal cancer cells via the AKT pathway [9]. Here, we explored whether *Plek2* knockdown affects this pathway in LLC cells. Western blot analysis revealed that *Plek2* knockdown reduced levels of AKT and phosphorylated AKT at serine 473 (Fig. 8d), indicating an association between *Plek2* knockdown and AKT pathway inhibition.

To investigate the impact of *Plek2* on immunotherapy efficacy, we implanted 5 million *Plek2* knockdown or 3 million *Plek2*-overexpressing LLC cells into the backs of immunocompetent C57BL/6 mice. On days 7, 10, and 13 after tumor implantation, we administered isotype control or PD-1 antibodies intraperitoneally. Under immunocompetent conditions, consistent with prior in vivo experiments, *Plek2* knockdown continued to inhibit tumor growth (Fig. 8e). Notably, the combination of *Plek2* knockdown and PD-1 antibody treatment significantly suppressed tumor growth rates and weights compared to controls (Fig. 8i–k). In contrast, in the *Plek2* overexpression group that received PD-1 treatment, tumor growth rates and weights increased, though not statistically significantly (Fig. 8f). These results suggested that *Plek2* knockdown sensitized LLC tumors to PD-1 immunotherapy, while *Plek2* overexpression induce resistance to PD-1 treatment.

To explore the correlation between *Plek2* knockdown and immunomodulatory molecules, we collected tumor tissues from C57BL/6 mice bearing tumors and extracted RNA for RT-PCR analysis of Cd276, Cd274, and Lgals9 expression. Results showed a notable reduction in Cd276 expression in the *Plek2* knockdown group, particularly in the sh-2 group (Figure S7e). Although Cd274 and Lgals9 expression levels were slightly reduced, these changes were not statistically significant (Figures S7f and S7g). These findings suggested that *Plek2* knockdown might improve the immunosuppressive state within tumors.

Given that CD8 T cells are the primary responders to PD-1 antibody treatment, we used IHC to assess CD8α T cell infiltration within tumors. *Plek2* knockdown increased CD8α T cell infiltration (Fig. 8g, top panels; Fig. 8i), while *Plek2* overexpression reduced it (Fig. 8h, top panels; Fig. 8i). Based on our pan-cancer single-cell analysis (Fig. 2), we hypothesized that *Plek2* may also affect macrophage infiltration within tumors. IHC analysis showed that *Plek2* knockdown decreased F4/80-positive cell infiltration (Fig. 8g, bottom panels; Fig. 8j), whereas *Plek2* overexpression increased it (Fig. 8h, bottom panels; Fig. 8j).

In summary, *Plek2* knockdown enhanced CD8α T cell infiltration and reduced TAMs, promoting a more immunostimulatory environment. Conversely, *Plek2* overexpression fostered an immunosuppressive tumor microenvironment, likely contributing to resistance to PD-1 therapy. These findings suggested that targeting *Plek2* could enhance immunotherapy efficacy.

## Discussion

In this study, we comprehensively investigated the role of PLEK2 through a pan-cancer analysis. Our analyses revealed that *PLEK2* is overexpressed in multiple cancer types at both the mRNA and protein levels. Single-cell RNA sequencing data indicated that *PLEK2* expression is predominantly found in tumor cells and macrophages within the tumor microenvironment. Notably, high *PLEK2* expression is associated with poor prognosis in several cancers, although its impact varies across different cancer types. We also found that *PLEK2* expression correlates with immunosuppressive states within tumors and has the potential to predict immunotherapy efficacy in certain cancers. Functional experiments demonstrated that *PLEK2* knockdown suppressed tumor cell proliferation and migration both in vitro and in vivo. Furthermore, *Plek2* knockdown in an LLC-bearing mouse model enhanced the efficacy of PD-1 immunotherapy, highlighting its potential as a therapeutic target.

Our findings indicated that *PLEK2* played a significant role in tumor progression by promoting cell proliferation and migration. Knockdown of PLEK2 in multiple human cancer cell lines led to decreased proliferation and migration (Fig. 7). Specifically, in the LLC model, *Plek2* knockdown significantly reduced tumor growth rates and tumor weights in vivo (Fig. 8b-c). Mechanistically, our previous research in esophageal cancer cells confirmed that *PLEK2* knockdown inhibited tumor growth and metastasis by suppressing the AKT pathway [9]. Consistent with this, the current study showed that *Plek2* knockdown in LLC cells reduced both AKT and phosphorylated AKT at serine 473 (Fig. 8d). Given that the AKT pathway is crucial for cell survival, growth, and metabolism [32–34], this inhibition likely contributed to reduced tumor growth and migration.

Our comprehensive analysis revealed that PLEK2 expression was significantly associated with various immune regulatory genes across multiple cancer types, including CD276 (B7-H3), CD274 (PD-L1), and FAS (Fig. 5c). CD276 and CD274 are established markers known to affect CD8 T cell infiltration and functionality [35–38], while FAS regulates T-cell apoptosis, thereby limiting T-cell responses [39]. In our in vivo experiments, *Plek2* knockdown was associated with decreased expression of Cd276 (B7-H3), suggesting that PLEK2 might foster an immunosuppressive tumor microenvironment (TME).

Our findings suggested that PLEK2 influenced the TME in ways that directly affected PD-1 blockade efficacy. Bioinformatics analysis indicated a positive correlation between PLEK2 expression and macrophage infiltration, suggesting that PLEK2 might have promoted the recruitment or activation of tumor-associated macrophages (TAMs) (Fig. 2 and 5d). Single-cell analysis showed that PLEK2 was highly expressed in both macrophages and epithelial cells (Fig. 2a), with PLEK2 expression in epithelial cells potentially enhancing tumor cell proliferation and TME interactions. However, we focused on macrophages because (1) macrophages were among the most prominently expressing cell types in our pan-cancer analysis, aside from tumor cells, and (2) they play a critical role in shaping the immune landscape within tumors. Numerous studies have confirmed the importance of the TME in influencing responses to tumor immunotherapy [40, 41].

In our LLC mouse model, *Plek2* knockdown enhanced the efficacy of PD-1 antibodies, resulting in a more pronounced reduction in tumor growth (Fig. 8e). This suggested that PLEK2 inhibition may have alleviated immunosuppression, thereby enhancing anti-tumor immune responses. This therapeutic improvement appeared to stem from a shift in the immune cell landscape: in PD-1 Ab–treated mice, *Plek2* expression correlated negatively with CD8 T cell infiltration and positively with macrophage presence (Figs. 8g-j). This observation supported the hypothesis that PLEK2 facilitated macrophage recruitment, potentially at the expense of CD8 T cell activity, contributing to an immunosuppressive environment and resistance to PD-1 therapy. Reducing *Plek2* expression seemed to restore this balance, allowing for greater CD8 T cell infiltration and enhancing the anti-tumor effects of PD-1 blockade. Increasing evidence has indicated that M2 macrophages within TAMs contribute to an immunosuppressive environment that inhibits CD8 T cell activity, a key effector in PD-1 therapy [42–45]. Given that PLEK2 influences the AKT pathway, a critical regulator of cell survival and proliferation, we speculated that *Plek2* knockdown might similarly inhibit AKT signaling in M2 macrophages. Further experiments would be needed to explore this mechanism in detail.

The analyses of OS, PFS, DSS, and DFS revealed that PLEK2's expression was significantly linked to the prognosis of cancer patients (Fig. 3). Interestingly, while *PLEK2* emerged as a risk factor contributing to poorer outcomes in a vast array of cancers, it simultaneously served as a protective factor in specific cancer types, including BLCA, BRCA, COAD, KIRC, KIRP, LAML, PCPG, READ, THCA, and USC. These findings underscored the complex and dualistic nature of *PLEK2*'s role across different cancer contexts. Meanwhile, these findings suggested that its role as a biomarker was particularly significant in certain cancers, such as lung cancer and melanoma, where high *PLEK2* levels correlate strongly with poorer clinical outcomes and reduced immunotherapy response. These associations indicated that *PLEK2* could serve as a more precise biomarker in these cancers, aiding in the stratification of patients who might benefit from targeted therapies. Given the intricate relationships observed, future research endeavors should aim at unraveling these complex functions and delving into the underlying mechanisms by which *PLEK2* influences cancer prognosis, thus paving the way for targeted therapeutic strategies and precision medicine.

However, our research indicated a positive correlation between *PLEK2* expression and tumor mutation burden (TMB) in most cancers (Fig. 5b), which typically suggested that higher *PLEK2* expression could be associated with a more responsive tumor to immunotherapy. This observation seemed to contradict our previous speculations. This contradiction was possibly due to the complex role of PLEK2 in immunotherapy response, involving an interplay between enhancing mutation burden and promoting immune evasion strategies of the tumor. This dual role could make PLEK2 a more nuanced biomarker, where its overall impact on immunotherapy response might depend on the balance between these opposing effects [46, 47]. Further research was necessary to dissect these mechanisms and understand how PLEK2’s effects on TMB and the tumor microenvironment (TME) could be leveraged to improve immunotherapy outcomes.

To our knowledge, this study was the first comprehensive exploration of the significance of PLEK2 in tumor immunology on a pan-cancer scale, corroborating its functionality across various cancer cell lines. However, this research had certain limitations. Primarily, our pan-cancer findings were derived predominantly from the amalgamation of data across several databases. While this expansive approach provided broad insights, it was subject to potential systematic inaccuracies inherent in such analytical methodologies. However, such databases are not without limitations, including sample heterogeneity, batch effects, and data imbalance. To mitigate these biases, we applied normalization techniques to minimize technical variations and used multiple datasets to validate our findings. Additionally, we adjusted for potential confounding factors such as patient demographics and tumor stage during our analyses. Despite these measures, the retrospective nature of these datasets should be considered when interpreting our results. While our findings supported *PLEK2*’s role in tumor progression and immune modulation, further studies focusing on specific molecular mechanisms, such as the downstream effects of *PLEK2* on AKT pathway or its influence on macrophage polarization, would help solidify these causative links. Future research exploring these pathways could build on our findings and deepen the understanding of *PLEK2* as a potential therapeutic target.

In conclusion, our comprehensive pan-cancer analysis demonstrated that PLEK2 played a multifaceted role in cancer progression and immune modulation. Overexpression of PLEK2 promoted tumor growth and created an immunosuppressive microenvironment by modulating immune cell infiltration and immunosuppressive molecules. Targeting PLEK2 not only suppressed tumor proliferation and migration but also enhanced the efficacy of immunotherapy, particularly PD-1 blockade. These findings suggested that PLEK2 was a promising prognostic biomarker and therapeutic target in cancer. Incorporating PLEK2 status into clinical decision-making could optimize treatment strategies and improve patient outcomes.

## Materials and methods

### Data collection

Data encompassing genomic characteristics, and patient clinical histories from 33 tumor samples were gathered from both TCGA (https://portal.gdc.cancer.gov/) and the GTEx projects (https://gtexportal.org/home/). We incorporated data from four different immunotherapy research groups: the IMvigor210 study, focusing on kidney cancer patients treated with anti-PDL1; the GSE100797 study, involving melanoma patients undergoing adoptive T cell therapy; the study by VanAllen on metastatic melanoma treated with anti-CTLA-4; and the GSE78220 study on melanoma treated with anti-PD-1. The clinical outcomes and gene expression data for these groups are accessible through the Gene Expression Omnibus (GEO) database (http://www.ncbi.nlm.nih.gov/geo). Additionally, information on various cancer cell lines was retrieved from the Cancer Cell Line Encyclopedia (CCLE) (http://www.sites.broadinstitute.org/ccle). Furthermore, we acquired immunohistochemical imagery showcasing PLEK2 protein expression across diverse tissues from the Human Protein Atlas (HPA) (http://www.proteinatlas.org/). The single-cell expression levels of *PLEK2* in various cancer tissues were analyzed using the TISCH database (http://tisch.comp-genomics.org/home/). Single cell sequencing datasets for hepatocellular carcinoma (GSE125449), cervical cancer (GSE171894), stomach cancer (GSE183904), and esophageal cancer (GSE188900) were included.

### Single-cell expression analysis of *PLEK2*

The single-cell expression levels of *PLEK2* across various tumor tissues were examined using the TISCH database. Expression data of *PLEK2* mRNA from different cell types across 88 datasets were downloaded and visualized graphically. Principal component analysis was used for dimension reduction, and batch effects were removed using the Harmony R package. We employed the UMAP function for visualizing the reduced dimensions and used the Leiden algorithm to cluster all cells. Comprehensive visualization of *PLEK2* expression details was achieved using Vlnplot, Dimplot, and Featureplot methods.

### Tumor immune dysfunction and exclusion (TIDE) analysis

TIDE stands as a benchmark computational model designed to forecast the outcomes of immune checkpoint blockade therapies in cancer patients [19]. TIDE was designed to assess T cell dysfunction and exclusion in different tumor types using transcriptomic data, providing a comprehensive overview of the immune microenvironment and its impact on ICI responsiveness. The TIDE framework used transcriptomic data from over 33,000 tumor samples to assess immune dysfunction in “hot” tumors and T cell exclusion in “cold” tumors. It has shown superior predictive capabilities compared to traditional biomarkers like PD-L1 expression and tumor mutational burden [48]. It was particularly noted for its effectiveness in lung cancer and melanoma, where the TIDE score serves as a dependable marker for anticipating the success of treatments involving anti-PD-1/L1 or anti-CTLA-4. Cancer-specific transcriptomic data were submitted to the TIDE portal for evaluation (http://tide.dfci.harvard.edu). Utilizing the platform's analytical tools, TIDE scores were calculated and then retrieved for each patient, setting the stage for further investigative analysis.

### Analysis of immunotherapeutic responses

The evaluation of responses to immunotherapy was conducted in line with the RECIST V1.1 Criteria, categorizing outcomes as complete response (CR), partial response (PR), stable disease (SD), and progressive disease (PD). Patients who achieved CR or PR were grouped under responders, while those with SD or PD were considered non-responders. To analyze the variation in *PLEK2* expression between these groups, the Student's t-test was employed. Additionally, TISMO tumor immune network tool (http://tismo.cistrome.org/) was used to compare gene expression levels before and after PDL1 and CTLA-4 treatments in cell lines [29].

### Identification of immune characteristics

Tumor Mutation Burden (TMB) is defined as the cumulative count of genetic alterations within cancer cells, encompassing coding errors in somatic genes, insertions, deletions, and nucleotide substitutions, with this aggregate mutation count subsequently normalized by the genome's exome size. For each tumor sample analyzed, TMB was determined using a reference exome size of 38 Mb. Microsatellite Instability (MSI) scores for the TCGA samples were sourced from existing scholarly publications. Following this, we explored the relationship between *PLEK2*'s expression levels, TMB, and MSI scores. The EPIC algorithm was employed to investigate how *PLEK2* interacts with immune cell populations [49].

### Gene Set Enrichment Analysis (GSEA)

Gene Set Enrichment Analysis (GSEA) facilitated the examination of differential signaling pathways between groups exhibiting high and low *PLEK2* expression, utilizing Kyoto Encyclopedia of Genes and Genomes (KEGG) for reference [50]. Analysis and graphical representation were conducted using R software (version 4.3.2), together with the following packages: limma for differential expression analysis, org.Hs.eg.db for gene annotation, clusterProfiler for statistical analysis and visualization of functional profiles, enrichplot for enriching visualization, and DOSE for disease ontology enrichment analysis.

### Drug sensitivity of *PLEK2* in the pan-cancer analysis

The compound activity data for the NCI-60 cancer cell lines, along with RNA sequencing data, were retrieved from the Genomics of Drug Sensitivity in Cancer (GDSC) databases (https://www.cancerrxgene.org/) [30]. Analysis focusing on the expression profiling and the investigation of *PLEK2*'s influence on drug sensitivity across various cancer types was conducted. For this purpose, the 'limma' package was utilized for differential expression analysis, while 'ggplot2' and 'ggpubr' R packages were employed to create and enhance the visualization of the results.

### Cell lines and cultures

To explore the role of *PLEK2*, nine cancer cell lines, sourced from ATCC, were selected. Information on *PLEK2* expression (Figure S8) and the origins of these cell lines were publicly accessible (https://depmap.org/portal/). The cell lines A549, H226, H460, KTSE-450, and H2052 were grown in RPMI 1640 medium (#10‐040‐CV, Corning), enriched with 10% fetal bovine serum (FBS) (#35‐081‐CV, Corning) and 100 U/ml penicillin–streptomycin. Meanwhile, MDA-MB-231, HCT116, PANC-1, UM-UC-3, and LLC cell lines were maintained in DMEM medium (#10‐013‐CVR, Corning), also supplemented with 10% FBS and 100 U/ml penicillin–streptomycin. These cells were all cultured under conditions of 5% CO_2_ at a temperature of 37 °C.

### siRNA reverse transfection assay

The siRNA duplexes were sourced from Generay Biotech in Shanghai, China, and introduced into cells using Lipofectamine 3000 reagent (L3000015, Invitrogen), strictly following the protocol provided by the manufacturer.

### Stable overexpression or knockdown of *Plek2* in LLC cell line

The shRNA expression vector targeting *Plek2* and the scrambled shRNA non-target control were procured from GenePharma. The *Plek2* lentiviral overexpression vector and the empty vector were obtained from Beijing Syngentech Corporation (Beijing, China).

### Quantitative real-time polymerase chain reaction (qRT-PCR)

RNA extraction was performed using the RNA-Quick Purification Kit (Catalog No. ES-RN001), followed by cDNA synthesis through the TransScript II All-in-One First-Strand cDNA Synthesis SuperMix for qPCR (Product No. AH341 from TransGen). The expression levels of various genes were then assessed via qRT-PCR, employing the SYBR Green protocol. This analysis was conducted on a 7900 Real-Time PCR System, utilizing the SYBR™ Select Master Mix for optimal detection.

### Western blotting analysis

Proteins were extracted using RIPA lysis buffer supplemented with protease inhibitors. The concentration of the resulting cell lysates was determined through a BCA protein assay. Proteins, in equal amounts, were then subjected to separation via 10% SDS-PAGE and subsequently transferred onto PVDF membranes. To block non-specific binding, the membranes were incubated in 5% skim milk. After blocking, the membranes were treated with various primary antibodies overnight at 4 °C. This was followed by incubation with HRP-conjugated secondary antibodies for a minimum of two hours. Post-incubation, the membranes were washed, and the protein signals were visualized using electrochemiluminescence detection methods. The following antibodies were used: anti-PLEK2 (Rabbit, 11685–1-AP, Proteintech), anti-α-Tubulin (Rabbit, T9026, Sigma), anti-Akt (Rabbit, 9272, CST), anti-p-Akt (Ser 473) (Rabbit, 4060, CST).

### Cell proliferation detection

The Cell Counting Kit-8 (CCK-8, Dojindo, Catalog No. CK04) was utilized for cell viability assays. Initially, 1.5 × 10^3^ cells were seeded in each well of 96-well plates and cultured under previously specified conditions. At designated time points (0 h, 24 h, 48 h, 72 h, and 96 h), a mixture of 10 μl CCK-8 solution and 90 μl of the culture medium was added to each well. Following a 2-h incubation period at 37 °C, the absorbance of each well was measured at a wavelength of 450 nm to determine cell viability.

### Cell migration assay

An 8.0 μm pore size transwell chamber was selected for assessing cell migration. Pan-cancer cells were enzymatically dissociated and resuspended in a serum-free medium. Subsequently, 5 × 10^4^ cells were carefully seeded into the upper chamber. The lower chamber was supplemented with medium containing 20% FBS to act as a chemoattractant. After 24 h, cells that had migrated through the pores to the lower chamber were fixed, stained, and visualized under a microscope for photographic documentation.

### Animal experiments

The study protocol was reviewed and approved by the Animal Care and Use Committee at the Cancer Hospital of the Chinese Academy of Medical Sciences (approved number: NCC2023C-567) and complied with the Declaration of Helsinski. A subcutaneous tumor model was generated by injecting 2 × 10^6^ LLC cells into BALB/c nude mice. For C57BL/6 mice, groups with *Plek2* knockdown or overexpression were established by implanting 3 × 10^6^ or 5 × 10^6^ LLC cells, respectively. Treatment began when the tumors in C57BL/6 mice reached an approximate volume of 50 mm^3^, at which point the mice were randomly assigned to receive either IgG2a (200 μg per mouse, BE0089, BioXcell, Shanghai, China) or anti-PD1 antibody (200 μg per mouse, BE0146, BioXcell, Shanghai, China) via intraperitoneal injection. Tumor volume was determined using the formula: volume = (length × width^2^)/2. Investigators blinded to the group assignments measured the tumor volume every 3 days, and growth curves were plotted based on the gathered data. The excised tumors were weighed and collected for further analysis.

### Immunohistochemistry study

LLC tumors were immersed in 10% neutral-buffered formalin for 24 h, followed by overnight permeabilization in 70% ethanol. The fixed tissues were then embedded in paraffin, sliced into sections, and placed onto slides for immunostaining with antibodies targeting mouse CD8α (Rabbit, 98,941, CST) and F4/80 (Rabbit, 70,076, CST).

### Statistical analysis

Statistical analyses within this research were executed using R (version 4.1.2) and GraphPad Prism software (version 9.5.0, California, USA). For comparisons between two groups, Student’s t-test was used after confirming normality (via Shapiro–Wilk test) and homogeneity of variances (using Levene’s test), whereas the Mann–Whitney U test was utilized for data not adhering to normal distribution. When comparing more than two groups, one-way ANOVA was applied, followed by post-hoc tests corrected with the Bonferroni method for multiple comparisons. Kaplan–Meier survival analysis and log-rank tests were used to assess the prognostic significance of *PLEK2* levels. The proportional hazards assumption for the Cox regression models was tested using Schoenfeld residuals. To control for multiple testing in the bioinformatics analysis, the Benjamini–Hochberg method was applied to adjust the false discovery rate (FDR), with an adjusted *P*-value < 0.05 considered significant. Correlations between variables were assessed using Spearman’s correlation for non-parametric data and Pearson’s correlation for parametric data.

## Supplementary Information

Supplementary Material 1.

Supplementary Material 2.

## Data Availability

The data and materials that support the findings of this study are available from the corresponding author J.H. upon reasonable request.

## Abbreviations

ACC: Adrenocortical cancer
ATCC: American Type Culture Collection
BLCA: Bladder cancer
BRCA: Breast cancer
CAF: Carcinoma-associated fibroblast
CESC: Cervical cancer
CHOL: Bile duct cancer
COAD: Colon cancer
DLBC: Large B-cell Lymphoma
DSS: Disease-specific survival
ESCA: Esophageal cancer
HNSC: Head and neck squamous cell carcinoma
HSC: Hematopoietic stem cells
GEO: Gene Expression Omnibus
GBM: Glioblastoma
GSEA: Gene Set Enrichment Analysis
GTEx: Genotype Tissue Expression Project
KICH: Kidney chromophobe
KIRC: Kidney clear cell carcinoma
KIRP: Kidney papillary cell carcinoma
IHC: Immunohistochemistry
LAML: Acute myeloid leukemia
LGG: Lower grade glioma
LIHC: Liver cancer
LUAD: Lung adenocarcinoma
LUSC: Lung squamous cell carcinoma
MESO: Mesothelioma
MSI: Microsatellite instability
NES: Normalized enrichment score
NSCLC: Non-small cell lung cancer
OS: Overall survival
OV: Ovarian cancer
PAAD: Pancreatic cancer
PCPG: Pheochromocytoma & paraganglioma
PLEK2: Pleckstrin-2
PRAD: Prostate cancer
PFS: Progression-free survival
READ: Rectal Cancer
SARC: Sarcoma
STAD: Stomach cancer
SKCM: Melanoma
TAM: Tumor associated macrophage
TCGA: The Cancer Genome Atlas
TGCT: Testicular cancer
THCA: Thyroid cancer
TISCH: Tumor immune single-cell hub
THYM: Thymoma
TMB: Tumor mutation burden
TME: Tumor immune microenvironment
TIMSO: Tumor Immune Syngeneic Mouse
UCEC: Endometrioid cancer
UCS: Uterine carcinosarcoma
UVM: Ocular melanomas
