# Supplementary material for: Unveiling the role of Pleckstrin-2 in tumor progression and immune modulation: insights from a comprehensive pan-cancer analysis with focus on lung cancer
Source: Mol Biomed. 2024 Nov 15;5:59. doi: 10.1186/s43556-024-00225-8 (PMC11568116; doi:10.1186/s43556-024-00225-8)
Supplement: Supplementary file 1 — Supplementary Material 1. [file 43556_2024_225_MOESM1_ESM.docx]

**Unveiling the Role of Pleckstrin-2 in Tumor Progression and Immune Modulation: Insights from a Comprehensive Pan-Cancer Analysis with Focus on Lung Cancer**

Enzhi Yin^1,2^*, Chengming Liu^1,2^*, Yuxin Yao^1,2^*, Yuejun Luo^1,2^*, Yaning Yang^3^, Xiaoya Tang^1,2^, Sufei Zheng^1,2^, Linyan Tian^3^, and Jie He^1,2^^#^

1.Department of Thoracic Surgery, National Cancer Center/National Clinical Research Center for Cancer/Cancer Hospital, Chinese Academy of Medical Sciences and Peking Union Medical College, 100021, Beijing, China

2.State Key Laboratory of Molecular Oncology, National Cancer Center/National Clinical Research Center for Cancer/Cancer Hospital, Chinese Academy of Medical Sciences and Peking Union Medical College, Beijing, China

3.Department of Medical Oncology, National Cancer Center/National Clinical Research Center for Cancer/Cancer Hospital, Chinese Academy of Medical Sciences and Peking Union Medical College, Beijing, China.

^*^Contribute equally to the article.

^#^Corresponding author: Jie He (prof.jiehe@gmail.com)

Contributing authors: Enzhi Yin: yinenzhi1987@163.com; Chengming Liu: ming3948@163.com; Yuxin Yao: yuxinyaotj@foxmail.com; Yuejun Luo: yuejunluo_daisy@163.com; Yaning Yang: yn_yang0815@163.com; Xiaoya Tang: xiaoyatang_tracy@163.com; Sufei Zheng: suphyzheng@163.com; Linyan Tian: Tly18846037830@outlook.com.

**Supplementary Methods**

**Data collection**

Normal tissue expression data was obtained from the GTEx database, while tumor data was sourced from TCGA. For each cancer type, normal tissues were selected to match the organ and tissue type of the corresponding cancer type. For instance, lung tissues from GTEx served as the normal comparison group for lung adenocarcinoma (LUAD) samples from TCGA. To account for differences in sample processing between TCGA and GTEx, we applied batch effect correction using the Combat function from the sva package in R. Quality control checks ensured that only samples with high RNA quality were included in the analysis, minimizing discrepancies between normal and tumor data. RNA-seq data from TCGA and GTEx were processed and normalized using TPM to adjust for differences in sequencing depth and gene length. To correct for batch effects between datasets, we used the Combat function in the sva package.

**The sequences for PLEK2 siRNA or shRNA used in this study**

The sequence of *PLEK2* siRNA is as follows: *PLEK2* siRNA-1: 5’-GCCUGGGCCUUUGAGAUCACCTT-3’; *PLEK2* siRNA-2: 5’-GGAUCCAGCUUUCCUGCAUTT-3’; Negative control siRNA: 5’-UUCUCCGAACGUGUCACGUTT-3’.

The sequences for the shRNAs against Plek2 are as follows: sh-1 is 5’-GGATCCAGCTTTCCTGCAT-3’, sh-2 is 5’-GGCTCATCTCCAACAGCTTCA-3’, and negative control is 5’-TTCTCCGAACGTGTCACGT-3’.

Primer sequences used in qRT-PCR are listed below: human GAPDH forward primer: 5’-GGACGAGATCCCTCCAAAAT-3’, reverse primer: 5’-GGCTGTTGTCATACTTCTCATGG-3’; PLEK2 forward primer: 5’-GATGCCTGGGCCTTTGAGAT-3’, reverse primer: 5’-ATGTTGGGGCTTGAACGGAT-3’; mouse GAPDH forward primer: 5’-AGGTCGGTGTGAACGGATTTG-3’, reverse primer: 5’-GGGGTCGTTGATGGCAACA-3’; mouse CD276 forward primer: 5’-AGCATCCAGGACTTTGACAGCG-3’, reverse primer: 5’-CGTGATGGTCACCATGTTCCCT-3’; mouse CD274 forward primer: 5’-TGCGGACTACAAGCGAATCACG-3’, reverse primer: 5’-CTCAGCTTCTGGATAACCCTCG-3’; mouse Lgals9 forward primer: 5’-CTGGAATCCCTCCTGTGGTGTA-3’; reverse primer: 5’-CCTCGTAGCATCTGGCAAGACA-3’.

**Animal experiments**

Female BALB/c nude mice (4 weeks old) and female C57BL/6 mice (4 weeks old) were obtained from the Institute of Hematology at the Chinese Academy of Medical Sciences (Tianjin, China). The mice were housed in an animal facility maintained under a 12-hour light-dark cycle at a temperature of 22°C in a specific pathogen-free (SPF) setting. Mice were euthanized using CO_2_ asphyxiation once a tumor reached a diameter exceeding 1.5 cm in any group.

**Supplementary Figures**

**
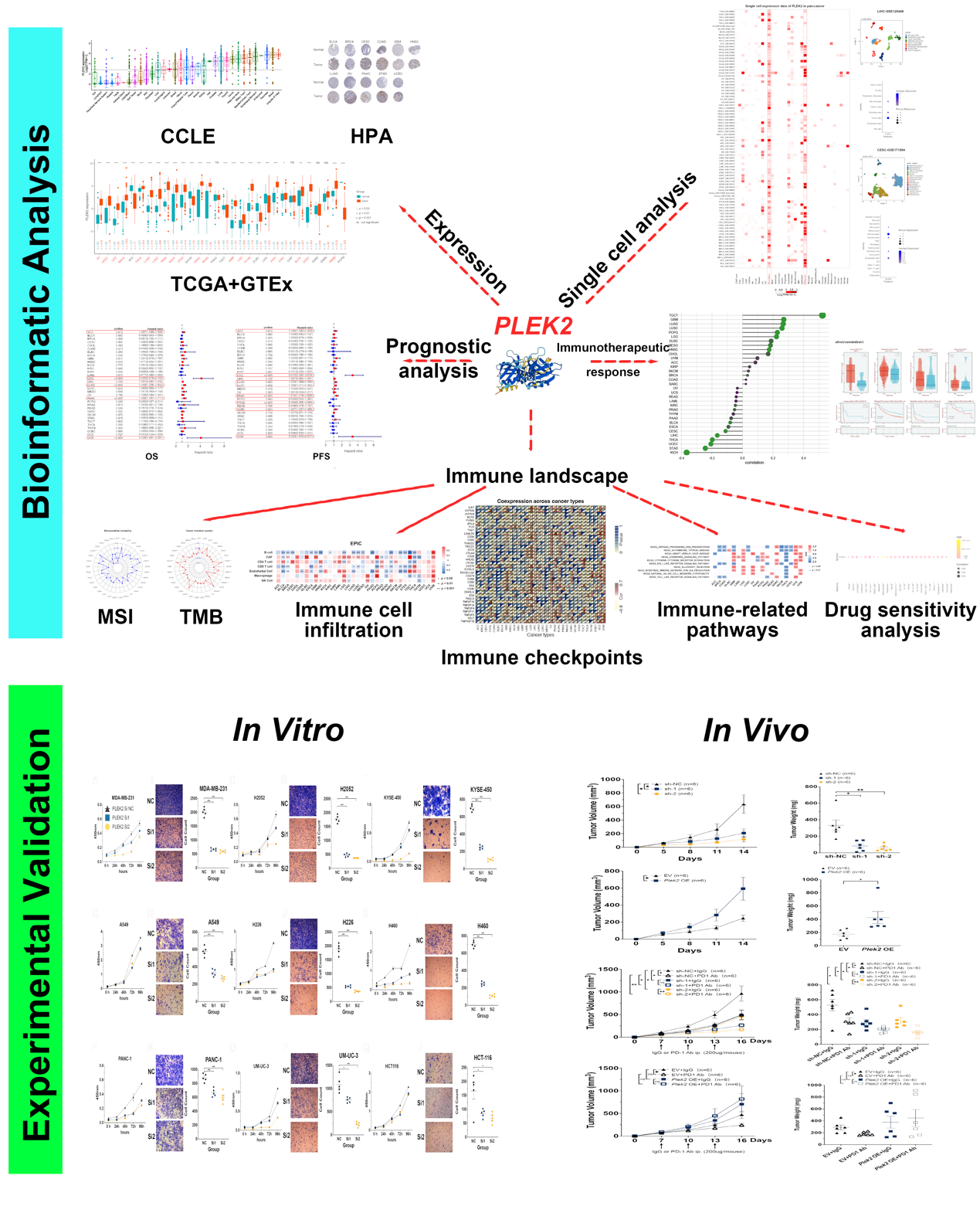
**

Figure S1. **Workflow and design of this study.**

**
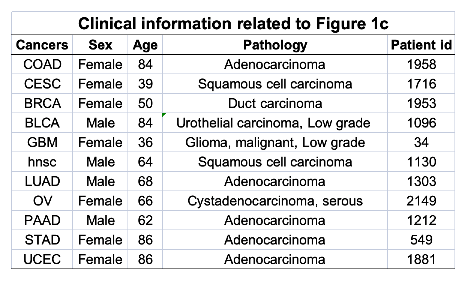
**

Figure S2. **Clinical information related to Figure 1c.**


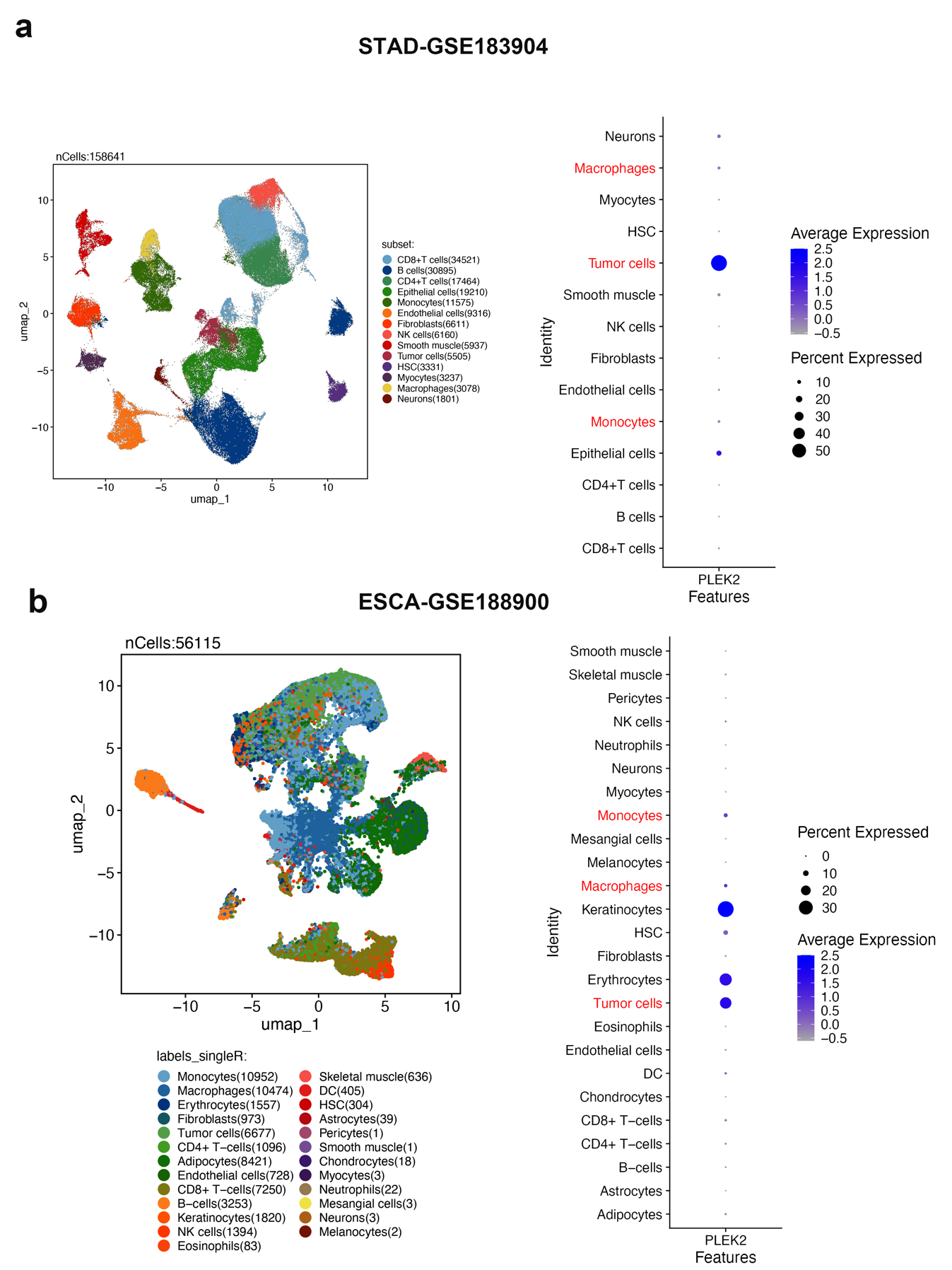


Figure S3 **Single-cell expression analysis of *PLEK2*.**

Single-cell expression analysis of *PLEK2* in STAD (**a**) and ESCA (**b**).


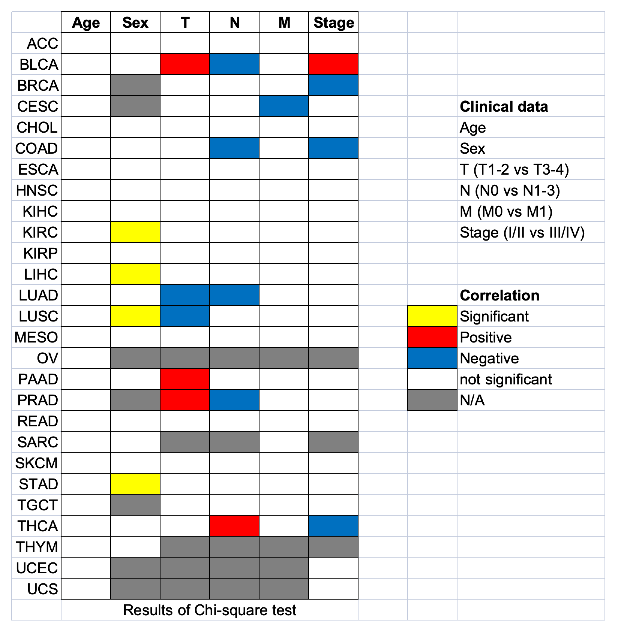


Figure S4 **Heatmap clusters showing the correlation between *PLEK2* expression levels and six clinical characteristics in 27 cancer types.**

Data from the TCGA database were obtained and analyzed using the Chi-square test. Yellow boxes indicate significant correlations with age or sex, while red and blue boxes reflect more advanced and low-grade clinical characteristics in patients with high *PLEK2* levels, respectively. White boxes denote non-significant outcomes, and gray boxes indicate unavailable data.


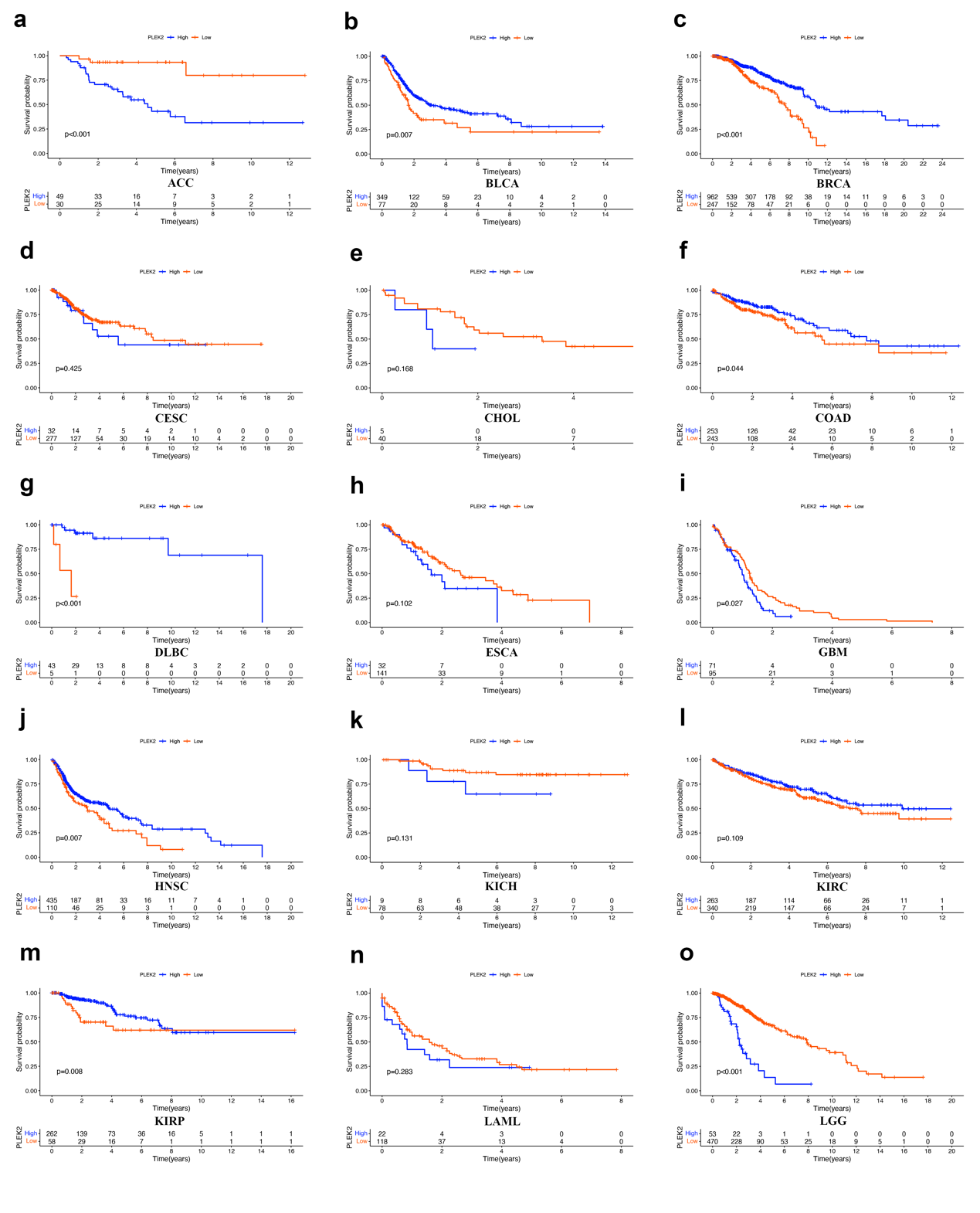


Figure S5 The Kaplan-Meier curves of OS in multiple cancers.

The Kaplan-Meier curves of OS (PLEK2 high vs. low) in ACC (**a**), BLCA (**b**), BRCA (**c**), CESC (**d**), CHOL (**e**), COAD (**f**), DLBC (**g**), ESCA (**h**), GBM (**i**), HNSC (**j**), KICH (**k**), KIRC (**l**), KIRP (**m**). LAML (**n**), LGG (**o**).


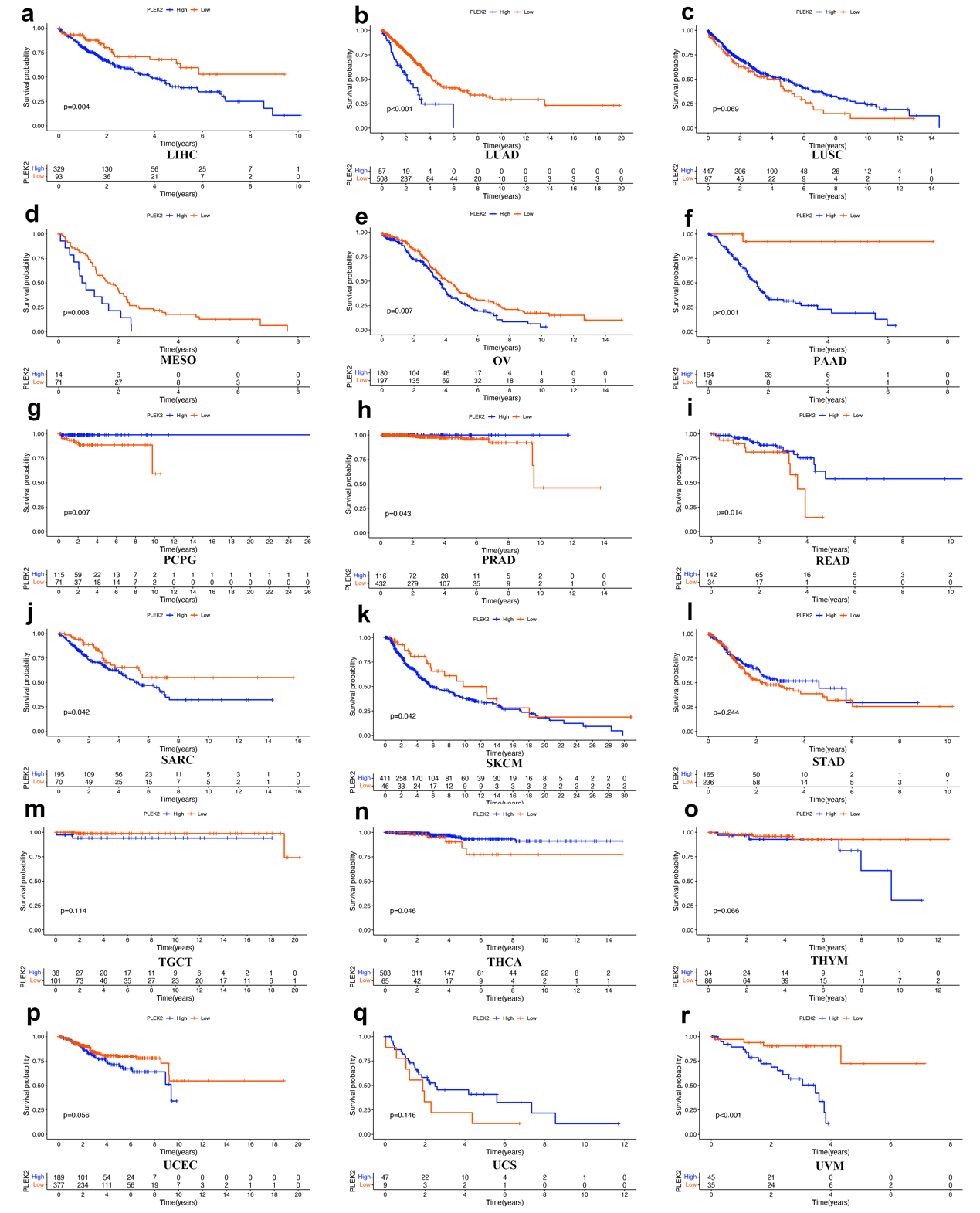


Figure S6 The Kaplan-Meier curves of OS in multiple cancers.

The Kaplan-Meier curves of OS (PLEK2 high vs. low) in LIHC (**a**), LUAD (**b**), LUSC (**c**), MESO (**d**), OV (**e**), PAAD (**f**), PCPG (**g**), PRAD (**h**), READ (**i**), SARC (**j**), SKCM (**k**), STAD (**l**), TGCT (**m**). THCA (**n**), THYM (**o**), UCEC (**p**), UCS (**q**), UVM (**r**).


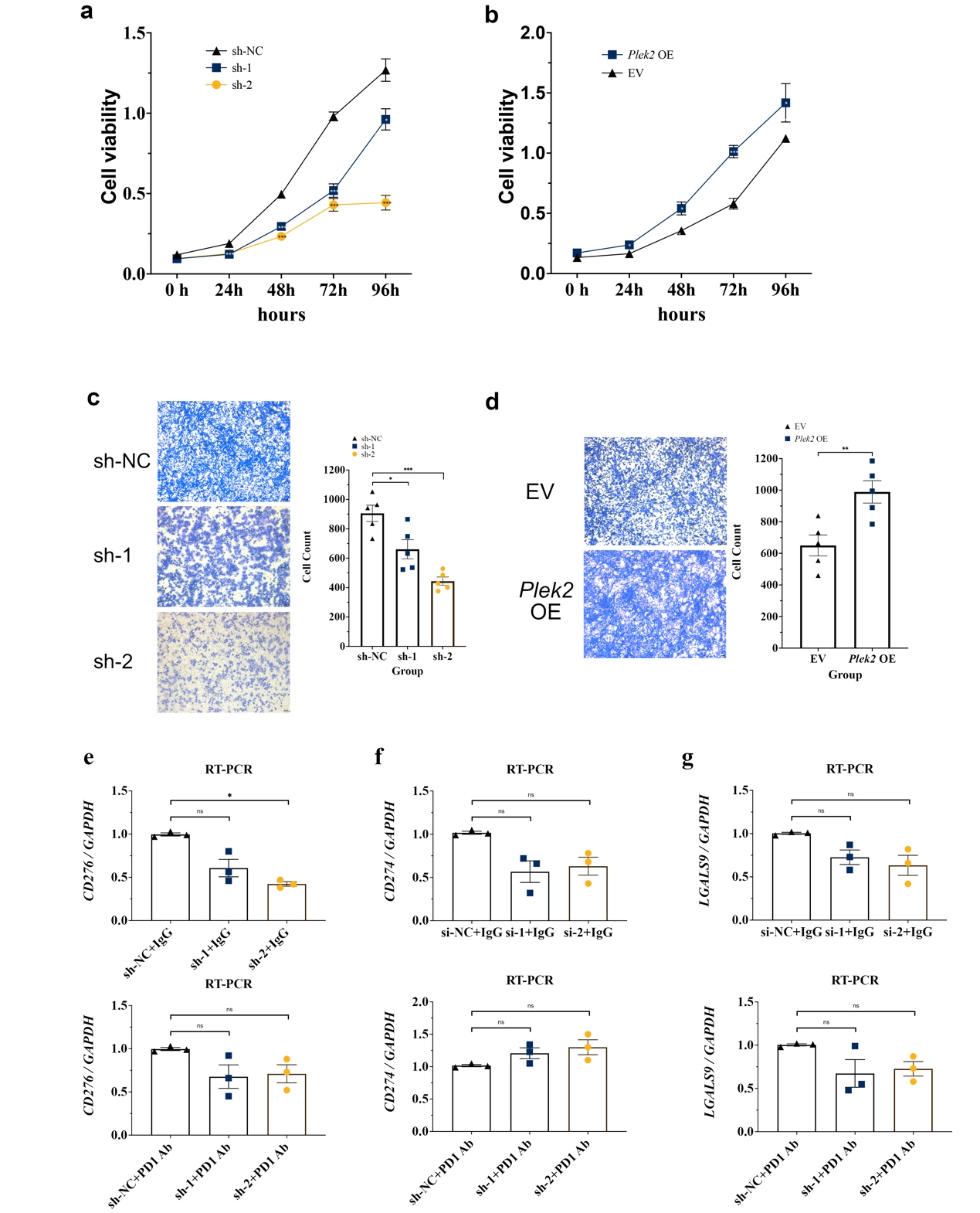


Figure S7 **Knockdown or overexpression of** ***Plek2* affects cell proliferation and migration on LLC cells.**

**A** Knockdown of *Plek2* inhibits cell proliferation compared with the sh-NC group; n=5/group. **b** Overexpression of *Plek2* promoted cell proliferation compared with the EV group; n=5/group. **c** Knockdown of *Plek2* inhibits cell migration compared with the sh-NC group; n=5/group. **d** Overexpression of *Plek2* promoted cell migration compared with the EV group; n=5/group. **e-g** Intratumoural RNA expression levels of indicated immunomodulatory molecules in C57BL/6 mice with (bottom panel) or without (top panel) PD1 antibody immunotherapy. n=3/group. Data were presented as mean ± SEM. Data were analyzed by one-way ANOVA.

**
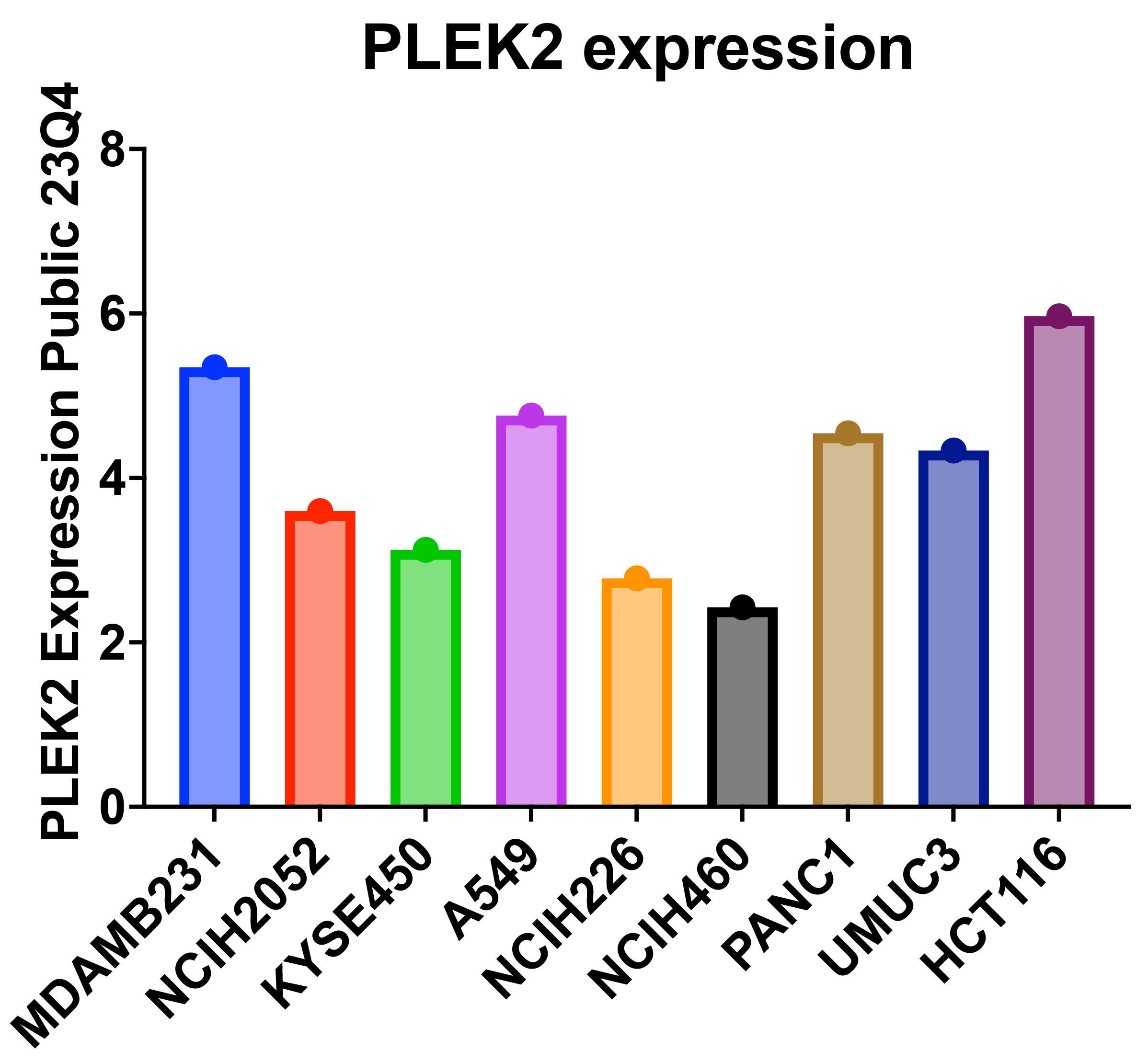
**

Figure S8. **Landscape of *PLEK2* expression data in MDA-MB-231, H2052, KYSE-450, A549, H226, H460, PANC-1, UM-UC-3 and HCT116 cells.**

Expression data are available online, at https://depmap.org/portal.

**Supplementary Table**

**Table S1. The clinical characteristics of the research samples.**

**It is provided separately, and attached as Excel files.**
